# Endoscopic Treatment of Stump Leakage Related to the Ileal Conduit

**DOI:** 10.1089/cren.2016.0042

**Published:** 2016-05-01

**Authors:** Bulent Odemis, Erkin Oztas, Muhammet Yener Akpinar, Erkan Olcucuoglu, Ertugrul Kayacetin

**Affiliations:** ^1^ Department of Gastroenterology, Türkiye Yüksek İhtisas Training and Research Hospital, Ankara, Turkey.; ^2^ Department of Urology, Türkiye Yüksek İhtisas Training and Research Hospital, Ankara, Turkey.

## Abstract

***Background:*** Ileal conduit with leakage from either the anastomotic site or the stump is associated with high morbidity and mortality rates. The standard treatment of stump leakage is surgery.

***Case Presentation:*** A 60-year-old male patient was admitted to our hospital with complaint of hematuria and bladder carcinoma was diagnosed. After performing radical cystectomy and ileal conduit, he developed fever with abdominal pain within the first week of surgery. Stump leakage was diagnosed by endoscopic examination performed through a gastroscope. After two over-the-scope clips (OTSCs) were applied to the stump, vinyl mesh was inserted into the space between the OTSCs. Later, cyanoacrylat and lipiodol were repelled on the OTSCs and vinyl mesh. Subsequently, stump leakage was resolved.

***Conclusion:*** This is the first case of stump leakage related to ileal conduit that has been treated endoscopically, according to the current literature.

## Introduction and Background

The ileal conduit technique is a type of urinary diversion commonly used after cystectomy. A short segment of ileum above the ileocecal valve resected and this segment is used for the passage of the urine across the abdominal wall to a cutaneous stoma.^1^ Different complications can be observed. Stump leakage is one of them that can lead to serious morbidity and mortality rates.

The over-the-scope clip (OTSC) for flexible endoscopy is a super elastic Nitinol device for compression and approximation of tissue in the human digestive tract.^2^ Cyanoacrylates can be used as rescue therapy for leaks from the bile or pancreatic duct when standard endoscopic therapy such as stenting has failed.^3^

Here we presented a patient having ileal conduit with stump leakage treated endoscopically by the combination of OTSC, cyanoacrylate, and vinyl mesh application.

## Case Presentation

**FIG. 1.**
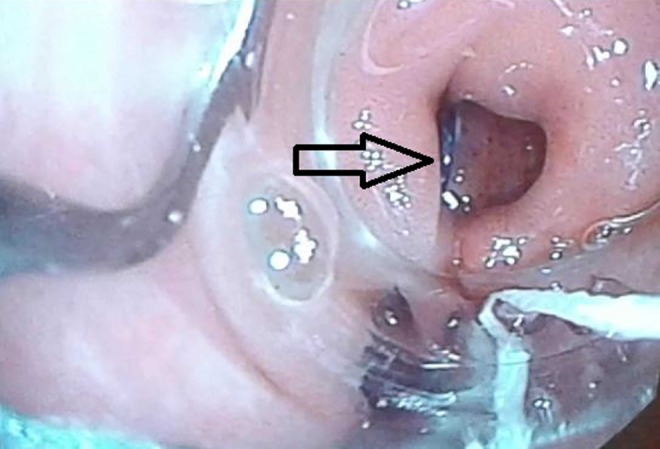
Gap at the stump of ileal conduit.

**FIG. 2.**
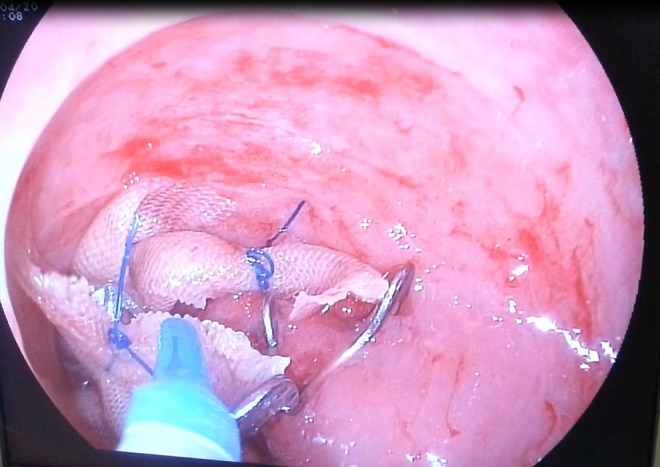
Mesh was placed between over-the-scope clips.

A 60-year-old male patient was admitted to our hospital with the complaint of hematuria. Bladder carcinoma was diagnosed in the patient. After having radical cystectomy and ileal conduit surgeries, he developed abdominal pain and fever within the first week of surgery. Laboratory tests revealed high-level of C-reactive protein, sedimentation rate, and leukocytosis. Pelvic fluid was seen in transabdominal ultrasonography. Subsequently, leakage possibility was considered because of ileal conduit. Anastomotic or stump leakage was suspected in cystography. Endoscopic examination was performed with standard gastroscope through cutaneous anastomotic orifice of ileal conduit. Ureteral anastomotic regions were intact. However, a large defect was seen at the stump (Fig. 1). First of all, one OTSC (Ovesco Endoscopy AG, Tubingen, Germany) was applied by using gastroscope. However, the leakage was not resolved because of continuous urine flow. In the second attempt, two more OTSCs were applied to the stump. Then, vinyl mesh was inserted into the space among OTSCs. At the end, cyanoacrylate and lipiodol were repelled on the OTSCs and vinyl mesh (Fig. 2). Leakage was completely resolved in the second week.

## Conclusion

The gold standard treatment of muscle invasive bladder cancer is radical cystectomy. The ileal conduit technique is a type of urinary diversion commonly used after radical cystectomy. For many years, it has been the leading urinary diversion method. Both early and late complications can be seen as a result of ileal conduit. Early complications such as bleeding, intestinal obstruction, anastomotic leakage, and urinary reflux can be seen in up to 48% of patients.^4,5^ Paralytic ileus and intestinal anastomotic leakage are well-known bowel-related early complications.^6^ Stump leakage is a rare condition. Despite its rare frequency, it is a serious complication. Standard treatment of stump leakage is surgery. The knowledge regarding nonsurgical approach and treatment of stump leakage is limited according to the literature. Endoscopic evaluation of ureteral orifices and stump can be used to diagnose some kind of leakage after surgery. Also, endoscopic treatment methods, for example, OTSC, hemoclips, or tissue adhesive can be applied when any kind of leakage was detected.

This is the first case of stump leakage related to ileal conduit, which has been treated endoscopically, according to the current literature. To our knowledge, no systematic research exists that addresses the treatment of stump leakage related to ileal conduit.

## Abbreviation Used

OTSC: over-the-scope clip
